# Subcritical co-solvents extraction of lipid from wet microalgae pastes of *Nannochloropsis* sp

**DOI:** 10.1002/ejlt.201100120

**Published:** 2012-02

**Authors:** Min Chen, Tianzhong Liu, Xiaolin Chen, Lin Chen, Wei Zhang, Junfeng Wang, Lili Gao, Yu Chen, Xiaowei Peng

**Affiliations:** Key Laboratory of Biofuels, Institute of BioEnergy and Bioprocess Technology, Chinese Academy of SciencesQingdao, China

**Keywords:** Extraction, Lipid, *Nannochloropsis* sp., Subcritical co-solvents, Wet microalgae

## Abstract

In this paper subcritical co-solvents extraction (SCE) of algal lipid from wet pastes of *Nannochloropsis* sp. is examined. The influences of five operating parameters including the ratio between ethanol to hexane, the ratio of mixed solvents to algal biomass (dry weight), extraction temperature, pressure, and time were investigated. The determined optimum extraction conditions were 3:1 (hexane to ethanol ratio), 10:1 ratio (co-solvents to microalgae (dry weight) ratio), 90°C, 1.4 MPa, and 50 min, which could produce 88% recovery rate of the total lipids. In addition, electron micrographs of transmission electron microscopy (TEM) and scanning electron microscopy (SEM) were conducted to show that the algal cell presented shrunken, collapsed with some wrinkles and microholes after SCE extraction. The main composition of total lipids extracted under the optimum conditions was TAG which represented more than 80%. And the fatty acid profile of triglycerides revealed that C16:0 (35.67 ± 0.2%), C18:1 (26.84 ± 0.044%) and C16:1 (25.96 ± 0.011%) were dominant.

**Practical applications:**

The reported method could save energy consumption significantly through avoiding deep dewatering (for example drying). The composition of the extracted lipid is suitable for the production of high quality biodiesel.

## Introduction

The rate of depletion of fossil fuels and the effect of greenhouse gas emissions on global climate change are creating more and more interest in biofuels 1–4. Of all the feedstock for biofuels, microalgae has been recognized as the most promising alternative source due to its advantages including high growth rate, short growth time, high lipid production, and compatible to wastewater and flue gas. Among the marine microalgae, *Nannochloropsis* sp. is extensively cultured for larval feed in aquaculture for its high content of PUFA. For large-scale production of the microalgae, closed tubular photobioreactors are often used 5. However, currently, commercialization production of microalgae biofuels, especially algal biodiesel faces huge challenges which are the shortage of oleaginous algal biomass in large scale and the high cost of production processes. It necessitates the development of highly efficient cultivation technology to produce large amount of algal biomass in limited land area at low cost at first, and then inventing new technologies in downstream processing to solve algal harvesting, oil extraction, and biodiesel conversion efficiently and un-expensively.

Oil extraction from algal cells is an expensive process. Currently, most methods for algal oil extraction are derived from conventional oil extraction methods for oil-bearing crops. The solvent extraction, including chloroform/methanol, hexane, isopropanol, and petroleum ether, etc. 6–8, was used popularly. Supercritical carbon dioxide was also introduced for algal lipids extraction instead of toxic organic solvents 9–12. Other methods such as expelling, microwave/ultrasonic assisted extraction have been also used. However, almost all of the above extraction methods require dried algal powder with water content no more than 10%. It should be known that even the algal paste obtained by centrifugal dewatering contains more than 60% water. Therefore, the energy consumption for drying is dramatically high 13. Apart from this, extraction time, organic solvent usage and its recycling, especially the instrument investment and the feasibility to industrial scale-up are very important considerations.

Our previous research showed that subcritical ethanol extraction for lipids from wet paste of microalgae could increase the lipid yields (unpublished observation). This process included two steps of dewatering the wet microalgae paste by ethanol first and then extraction by subcritical ethanol from dewatered algal pastes. The problem is that the ethanol usage in the first step for dewatering is too high to cause high-energy consumption of ethanol recycling. Therefore, it is critical to look for a new extraction method that can save cost and increase the yield. Using a second solvent in liquid–liquid extraction is common in extraction of lipids and carotenoid, and the second solvent could increase the yield of extraction 14–16. Here we propose subcritical co-solvents (the mixture of hexane and ethanol) extraction method in order to save energy consumption and increase the extraction yield of lipids. Using this method lipids can be extracted directly from wet microalgal paste of *Nannochloropsis* sp. without any deep dewatering. The effect of various experimental parameters, including ratio between ethanol to hexane, phase ratio of solvent to algal biomass, temperature, pressure, and time, on extraction efficiency was evaluated in this study.

## Materials and methods

### Chemicals and reagents

Sterol ester (SE), TAG, 1,3-DAG, free sterol (FS), 1,2-DAG, monoglyceride (MAG), and FAME standards were obtained from Sigma Co. (USA). All other organic solvents (*n*-hexane, ethanol, chloroform, acetic acid, and benzene) were purchased from HuaDa Co. (Guangzhou, China) and of analytical grade.

### Microalgae cultivation and harvesting

The strain of *Nannochloropsis* sp. was gifted by Dr. Qiang Hu from Arizona State University Polytechnic Campus. The microalgae were grown in glass panels in a BG11 medium 17 with ambient air and 2% CO_2_ agitating the culture. The volume of each panel is approximately 80 L. The cultivations were carried out in summer outdoor with temperature around 15–28°C and illumination intensity around 400–2000 mol photon m^−2^ s^−1^.

After cultivation, the biomass was harvested by membrane filtration using polyvinylidene fluoride ultrafiltration membrane, and then centrifuged for 10 min at 6000 rpm to obtain microalgae pastes. The water content of the wet microalgae was measured about 65%. All the algal pastes were stored at −80°C in darkness until extraction.

### Extraction of lipids

#### Determination of the total lipids content for microalgae samples

The content of total lipids in microalgae samples was determined according to Bligh and Dyer's method 18. The extracted lipids were transferred into a clean tube and the solvents were blown away under a gentle stream of nitrogen at 65°C. Then they were dried under vacuum at 70°C for 3 h. The lipids obtained from each sample were weighted. The lipids content was calculated *c*_0_ (% dry weight) as the standard for the following calculation.

#### Subcritical co-solvents extraction (SCE)

**Figure 1:**
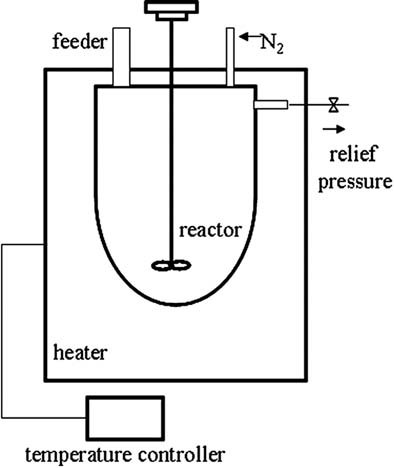
The schematic diagram of the apparatus for SCE.

**Figure 2:**
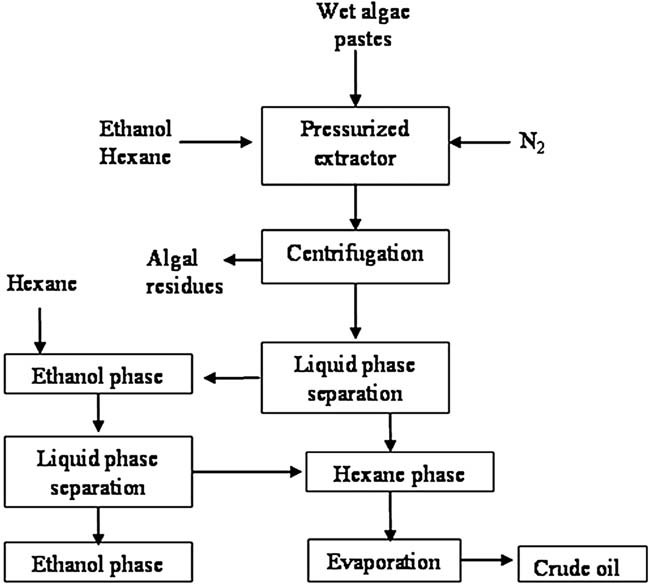
The process of subcritical co-solvent extraction of lipids from wet microalgae pastes of *Nannochloropsis* sp.

Subcritical co-solvents extraction (SCE) was performed with wet algae pastes using high-pressurized extractor (Xin Yuan Co., China) equipped with a stirrer and 100 mL stainless steel extraction cell. The diagram of the apparatus was shown in Fig. 1. The chamber had to be maintained at high pressure to prevent solvent from boiling during subcritical state extraction. The required pressure was achieved through the insertion of nitrogen gas into the chamber. The chamber was heated through oil bath, of which the temperature was controlled by a temperature controller. The extraction process consisted of the following steps as described in Fig. 2, (i) wet algae pastes and co-solvents (mixture of hexane and ethanol at a ratio) were inserted in the extraction chamber; (ii) N_2_ gas from a cylinder was accessed to keep the chamber's pressure; (iii) heating the chamber though oil bath to reach pre-set temperature; (iv) keeping stirring at 100 rpm/min during extraction; (v) cooling and depressurizing the system after pre-set extraction time; (vi) centrifuging the mixture, collecting ethanol phase and hexane phase; (vii) washing ethanol phase with hexane one time and collecting all hexane phase; (viii) evaporating hexane to obtain crude lipids.

The crude lipids were weighted and the recovery of the total lipids can be calculated by the following equation


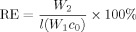


where, *W*_1_ is the dry weight of wet algal paste samples used, *c*_0_ the total lipid content of samples analyzed previously, and *W*_2_ is the total lipids extracted by SCE. Each extraction was repeated three times.

### Lipid composition analysis

#### The qualitative analysis of lipids

The components and content of extracted lipids were determined by modified thin-layer chromatography (TLC) method 19. The lipids were separated by TLC silica gel plates (50 mm × 100 mm in size) and were diffused by two mixed solvents. The first eluent was the mixture of methyl acetate, isopropanol, chloroform, methanol, and KCl (0.25% solution) in a ratio of 25:25:25:10:4 v/v/v/v running to a height of 7.5 cm from the origin. And the second eluent was the mixture of hexane, diethylether and acetic acid in a ratio of 70:30:2 v/v/v to a height of 9.5 cm from the origin. After dried, the plate was sprayed with iodine powder. Then individual lipid became detectable as yellow spot. Lipid components were identified by comparison with those of corresponding lipid standards.

For the recovery of individual lipid component, the corresponding silica gel was removed from the plates and washed it with chloroform/methanol (2:1 v/v). Individual TAG component was separated by TLC as described above, and kept for the fatty acid composition analysis as following.

#### The quantitative analysis of lipid by thin-layer chromatography with Iatroscan flame ionization detection (TLC-FID)

Three aliquots from total lipids were taken for lipid components separated by a Chromarods-S III (coated with silica). The separation of lipid components on the chromarods was performed in a two solvent systems: System I, Benzene/Chloroform/Acetic Acid (50:20:0.75 v/v); System II, Hexane/Benzene (35:35 v/v) 20. The separated lipids were identified by comparing their retain time with those of the respective standard samples. For the Iatroscan analyzer, the following conditions were used: air flow was 2000 mL/min; hydrogen flow was 160 mL/min; scan speed was 30 s/scan.

#### Fatty acid compositions analysis of TAG by GC

The fatty acid compositions analysis was performed by GC (Varian GC-450, US) according to Bigogno et al. 21. The TAG after qualitative analysis by TLC was transmethylated with 2% H_2_SO_4_ in methanol at 70°C for 1 h and C19:0 was added as an internal standard. The components were identified by comparing their retention times and fragmentation patterns with those of standards 22.

### The morphology of the original algae cell and the residue after reaction

#### The transmission electron microscopy (TEM)

The sample preparation was finished according to the methods mentioned by Liu and Lin 23. It consisted of the following steps: collecting the algal cells; fixing with 1% v/v glutaraldehyde and postfixing with 1% v/v osmium tetroxide both in sterilizing seawater; dehydration in a series of acetone solutions; suspension in the mixture of epoxy resin (Epon812) and acetone; embedded in 100% Epon812; polymerized and sectioned using a LeicaUC6 ultra microtome; picked up on 200-mesh copper grids and post-stained with urinal acetate. Finally, the sections were examined under a transmission electron microscopy (TEM; HITACHI H-7650) at an accelerating voltage of 60 kV.

#### The scanning electron microscopy (SEM)

The sample preparation for the scanning electron microscopy (SEM) was conducted as following steps: collecting the algal cells and washing the cells with sterilizing seawater for three times; fixing with 2.5% v/v glutaraldehyde for 1 h and postfixing with 1% v/v osmium tetroxide; rinsed with sterilizing seawater for 10 min for three times; dehydration in a series of 30, 50, 70, and 90% ethanol; suspended in the mixture of ethanol and tert-butyl alcohol and submerged in 100% tert-butyl alcohol; dried on critical point of tert-butyl alcohol. The dried sample was sputter-coated with platinum and examined with a SEM (HITACHI S-4800).

## Results and discussion

### The operating parameters of subcritical co-solvents extraction on the recovery of total lipids

#### The effect of hexane and ethanol ratio on the recovery of total lipids

**Figure 3:**
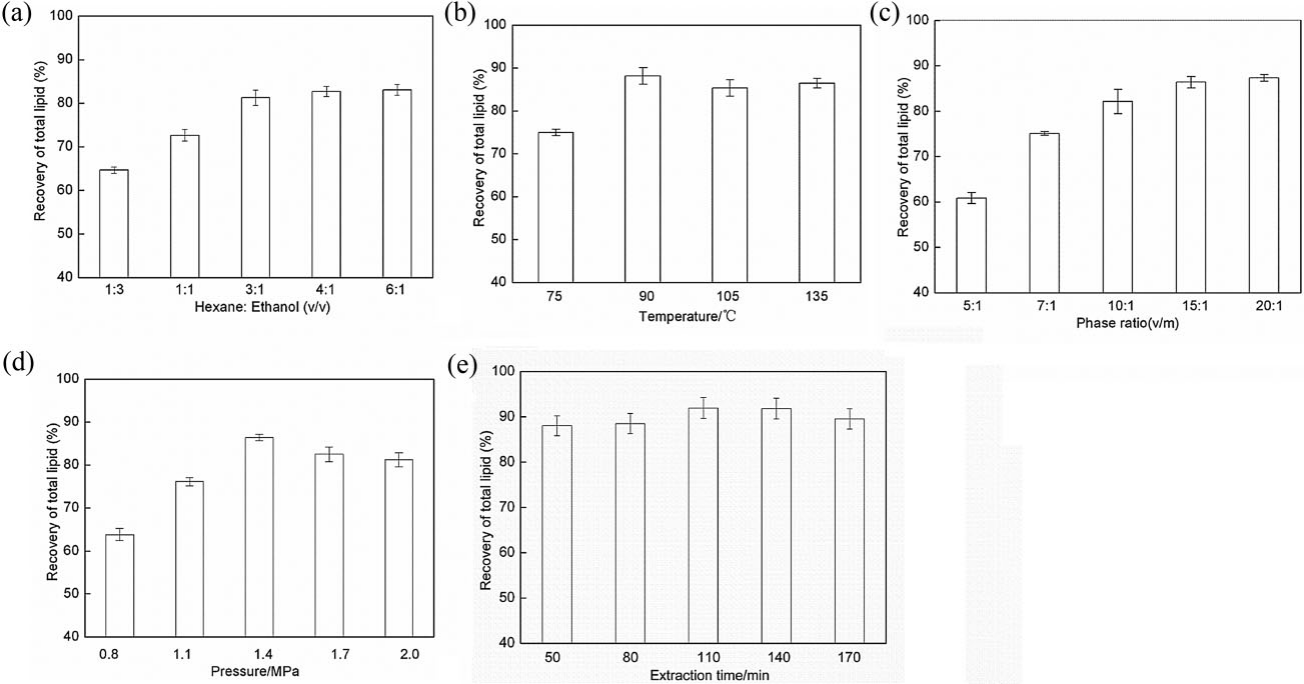
The operating parameters of SCE on the recovery of total lipids. (a) Effects of solvent ratio of hexane to ethanol on the recovery of total lipids (extraction conditions: phase ratio of co-solvents to dry algae: 10:1 v/m, temperature: 105°C, extraction time: 80 min, extraction pressure: 1.4 MPa, the total lipid content of the sample (dry weight) is 43.50%). (b) Effects of extraction temperature on the recovery of total lipids (extraction conditions: hexane to ethanol ratio: 3:1 v/v, phase ratio of co-solvents to dry algae: 10:1 v/m, extraction pressure: 1.4 MPa and extraction time: 80 min, the total lipid content of the sample is 43.50%). (c) Effects of the phase ratio on the recovery of total lipids between solvent mixtures to microalgae (dry weight) (extraction conditions: hexane to ethanol ratio: 3:1 v/v, temperature: 90°C, extraction pressure: 1.4 MPa and extraction time: 80 min, the total lipid content of the sample is 43.50%). (d) Effects of extraction pressure on the recovery of total lipids (extraction conditions: hexane to ethanol ratio: 3:1 v/v, phase ratio of co-solvents to dry algae: 10:1 v/m, temperature: 90°C and extraction time: 80 min, the total lipid content of the sample is 40.00%). (e) Effects of extraction time on the recovery of total lipids (extraction conditions: hexane to ethanol ratio: 3:1 v/v, phase ratio of co-solvents to dry algae: 10:1 v/m, temperature: 90°C and extraction pressure: 1.4 MPa, the total lipid content of the sample is 40.00%).

In order to evaluate the effect of the solvent ratio between hexane and ethanol ratio v/v on the recovery of total lipids, five ratios (1:3, 1:1, 3:1, 4:1, and 6:1) were examined to extract lipids. As shown in Fig. 3a, the results presented that hexane to ethanol ratio affected the recovery of total lipids. When the ratio of hexane to ethanol increased from 1:3 to 3:1, the recovery of total lipids rose from 65% to above 80%, but little increment was observed when the ratio further increased to 4:1 and 6:1. It is known that hexane is a non-polar solvent which has much higher solubility for lipid oil (mainly non-polar TAG) than ethanol even under subcritical status 24. During extraction, ethanol molecules entered into algal cell and extracted intra-cellular lipids and then backed to solvent bulk. However, ethanol is easy to saturate due to its lower solubility to lipids. Under the stirring, ethanol molecules will easily contact with hexane molecules in bulk and then the transference of lipids from ethanol to hexane takes place. As a result, ethanol in bulk would not be saturated by lipids. Thus, the adding of second solvent, hexane, promoted the extraction efficiency of ethanol. In this view, ethanol acted as a lipid carrier between algal cells and hexane. However, the driving force of mass transfer of lipids from ethanol to hexane is dominated by the difference of the content of lipids in hexane and the lipid solubility of hexane. More hexane in ethanol–hexane mixtures would dilute lipids in hexane and cause higher driving force of mass transfer. Thus increasing the ratio of hexane to ethanol increases the lipid recovery. But if hexane ratio is too high, the lipid carriers by ethanol would be not enough to promptly extract the lipid oil from algal cells. From the experimental results, hexane to ethanol ratio of 3:1 seemed appropriate.

#### The effect of extraction temperature on the recovery of total lipids

The effect of extraction temperature on the recovery of lipids was shown in Fig. 3b. It can be observed that an increase of extraction temperature enhanced lipid recovery. At 75°C, the recovery of lipid yield was <75%, however at 90°C, the recovery could be over 90%. This was probably because of the decrease of the viscosity of solvent and the increase of the lipid solubility in both ethanol and hexane at higher temperature 25. While the temperature rose to 105 and 135°C, the yield has a little decrease to 81.31 and 81.80%. Based on the results, the optimal temperature is suggested to be around 90°C.

#### The effect of the phase ratio on the recovery of total lipids between solvent mixtures to microalgae (dry weight)

Figure 3c shows the effect of the phase ratio between mixture solvent to microalgae (dry weight) on the recovery of total lipids. As can be seen in Fig. 3c, by raising the ratio from 5:1 to 10:1, the lipid recovery changed dramatically from 55.90 to 88.20%, indicating the significant influence of phase ratio of solvent to biomass. This finding was anticipated as increasing phase ratio improved contact and mass transfer between solvent and algal cells 26. However, further increase of the phase ratio from 10:1 to 20:1 improves the lipid recovery little increment. From economic view, phase ratio of 10:1 was appropriate.

#### The effect of extraction pressure on the recovery of total lipids

The effect of pressure on the extraction performance was investigated and displayed in Fig. 3d. It can be seen from Fig. 3d, at lower pressure of 0.8 MPa, the extraction recovery was definitely very low to about 60%. As mentioned above, ethanol is a polar solvent, its solubility to lipid, especially to TAG is very low at conventional temperature and pressure. Only when it was under subcritical status, its polarity reduced and could dissolve more lipids. Thus when the pressure increased from 0.8 to 1.4 MPa, ethanol would be more easy to enter microalgae cell to extract lipid and then transfer the lipid to hexane 27. But excessively high pressure had little effect on the lipid recovery. Obviously, the optimum pressure was 1.4 MPa.

#### The effect of extraction time on the recovery of total lipids

To evaluate the influence of extraction time on the extraction efficiency, lipid extractions were conducted for different times from 50 to 170 min at the above optimum operating parameters. Here the extraction time included the heating time of the extractor, extraction time, and cooling time. For the extraction process, the heating time from ambient to 90°C is about 25 min, and the cooling time is about 25 min to ambient. From Fig. 3e, it can be found that only slight variation when the total extraction increased from 50 to 170 min. It means that the extraction under subcritical conditions is a fast process. Longer time could not enhance the lipid recovery. It is difficult to conduct extraction experiments under much shorter time than 50 min because the temperature need be reduced to be lower than the boiling point of the co-solvent, and then the solvent won't boil after the pressure releasing. Therefore the sample could not be swiftly removed from the extractor since it took about 25 min for the decreasing of the solvent temperature. From Fig. 3e, it may be enough or better for extraction in a shorter time <50 min.

### Lipid composition analysis

#### The components analysis of extracted total lipid

**Figure 4:**
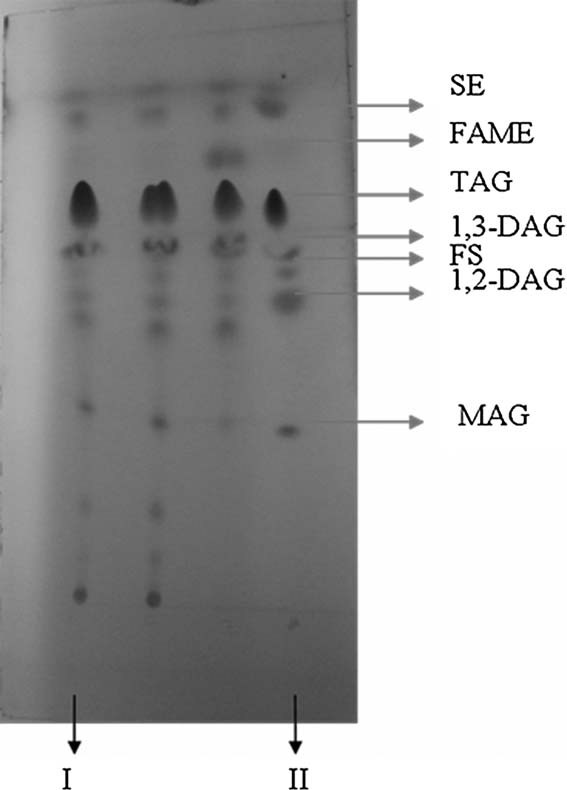
The qualitative analysis of lipid extracted by the optimum condition by TLC I represents the lipid extracted on the optimum condition; II represents the standards (SE, TAG, 1,3-DAG, FS, 1,2-DAG, MAG, and FAME).

**Figure 5:**
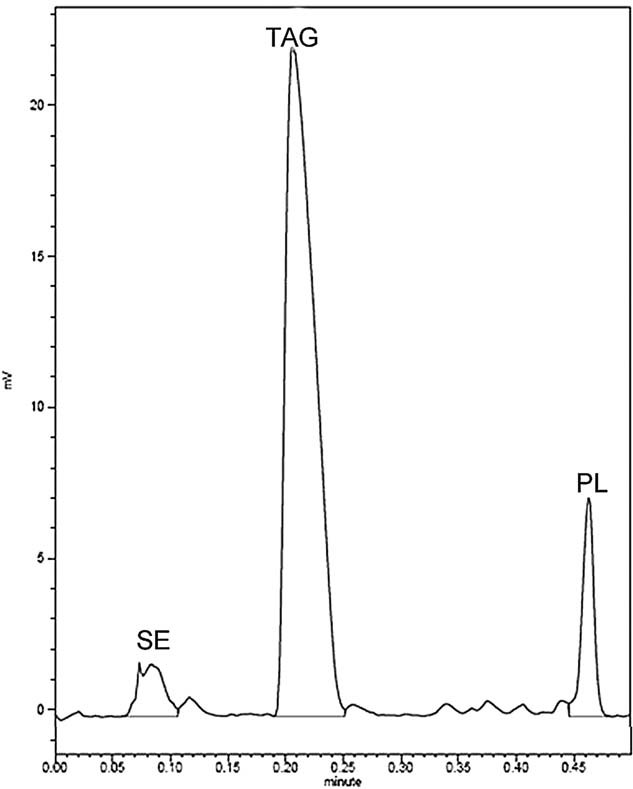
The quantitative analysis of lipid under the optimum conditions by TLC/FID.

The extracted lipids under the optimum conditions were subjected to TLC analysis. As shown in Fig. 4, lane (II) represented a mixture of standard lipids and was used to compare the *R*_f_ (ratio of front) values of the individual lipid components. Lane (I) corresponded to the extracted lipids by SCE under the optimum conditions. From the TLC results, it was confirmed that TAG, SE, 1,3-DAG, 1,2-DAG, MAG, and FS were the main components of the extracted lipids, but they contributed to the total lipid content in different amounts. As shown in Fig. 5, TAG made up an important percentage (more than 80%) of total lipids. SE represented <5% of total lipids and polar lipids (PL) were the other dominant fraction, mostly represented by glycolipids and phospholipids.

#### Fatty acid compositions of TAG

The major fatty acid composition of TAG was determined using a GC analysis. The results were showed in Table 1. It presented that C16:0, C18:1, and C16:1 were the dominants, about 35.67, 26.84, and 25.96%, respectively, and C20:5 (EPA), C22:6 (DHA), and other fatty acids were presented as fewer components, which was different from other literature 28. Oils with high oleic acid content have been reported to have a reasonable balance of fuel properties 29, 30. The properties of a biodiesel fuel, including its ignition quality, combustion heat, cold filter plugging point (CFPP), oxidative stability, viscosity, and lubricity, are determined by the structure of its component fatty esters. As such, a higher oleic acid (C18:1) content increases the oxidative stability for longer storage 31 and decreases the CFPP for use in cold regions 32. Therefore, the extracted total lipids are suitable for the production of good quality biodiesel.

**Table 1:** Fatty acid composition of TAG on the optimum condition

Fatty acid	Relative content (%)	Fatty acid	Relative content (%)
C14:0	2.88 ± 0.015	C20:0	0.14 ± 0.001
C16:0	35.67 ± 0.2	C20:5(EPA)	0.62 ± 0.02
C16:1	25.96 ± 0.011	C22:0	0.56 ± 0.001
C18:0	3.82 ± 0.013	C22:6(DHA)	0.36 ± 0.003
C18:1	26.84 ± 0.044	C24:0	0.32 ± 0.002
C18:2	0.80 ± 0.18	Undetermined	2.03 ± 0.004

### The morphology of original algae cell and the residue after reaction

**Figure 6:**
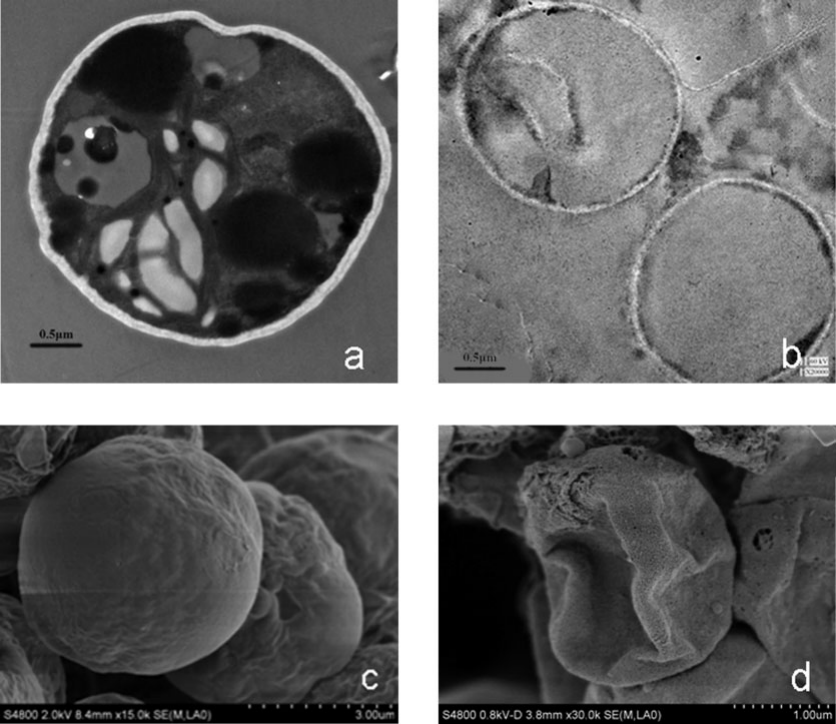
The morphology of original algae cell and the residue after reaction (a) TEM of the original algae cell of *Nannochloropsis* sp. (b) TEM of the residue extracted by SCE under the optimum conditions. (c) SEM of the original algae cell of *Nannochloropsis* sp. (d) SEM of the residue extracted by SCE under the optimum conditions. Scale bar: a = 0.5 µm; b = 0.5 µm; c = 3.0 µm; d = 1.0 µm.

Figure 6 illustrated the morphology of *Nannochloropsis* sp. and the extracted residues by SCE under the optimum conditions. In Fig. 6a, it can be seen that oil drops accumulated in the inner thylakoid spaces of the chloroplast structure. However, after extraction by subcritical co-solvents, as shown in Fig. 6b, the cell was almost completely emptied, indicating high extraction efficiency of SCE. Figure 6c presented the SEM image of an original *Nannochloropsis* sp. cell prior extraction, and Fig. 6d presented the SEM image of the cell after extracted by SCE. It can be found that after extraction, the algal cell shrunken, collapsed, and formed some wrinkles, and microholes. These results are identical with the results obtained by Ranjan et al. 33 who revealed that the mechanisms of lipid extraction by organic solvents is diffusion and disruption. The diffusion is due to the solvent and the disruption of cells is attributed to the friction of the solid with the stirring.

## Conclusions

Subcritical co-solvents were utilized to extract the total lipids from wet algae pastes of *Nannochloropsis* sp. The results showed that subcritical ethanol/hexane co-solvents could extract the total lipid from wet algal pastes efficiently. The optimum conditions were determined as follows: 3:1 hexane to ethanol ratio, 10:1 solvent to microalgae dry weight ratio, 90°C, 1.4 MPa, and 50 min. After the extraction, the algal cell shrank, collapsed with some wrinkles and microholes. The extracted lipid consisted of triglycerides above 80%, and the fatty acid composition of triglycerides revealed that C16:0, C18:1, and C16:1 was dominant, about 36, 27, and 26%, respectively.

## Abbreviations:

SCE: subcritical co-solvents extraction
SE: sterol ester
SEM: scanning electron microscopy
TEM: transmission electron microscopy
TLC: thin-layer chromatography
